# Self-harm to preferentially harm the pathogens within: non-specific stressors in innate immunity

**DOI:** 10.1098/rspb.2016.0266

**Published:** 2016-04-13

**Authors:** Edmund K. LeGrand, Judy D. Day

**Affiliations:** 1 Biomedical and Diagnostic Sciences, College of Veterinary Medicine, University of Tennessee, Knoxville, TN, USA; 2 Department of Mathematics and National Institute for Mathematical and Biological Synthesis, University of Tennessee, Knoxville, TN, USA

**Keywords:** host–pathogen interactions, physiological stress, infection, fever, anorexia, evolution

## Abstract

Therapies with increasing specificity against pathogens follow the immune system's evolutionary course in maximizing host defence while minimizing self-harm. Nevertheless, even completely non-specific stressors, such as reactive molecular species, heat, nutrient and oxygen deprivation, and acidity can be used to preferentially harm pathogens. Strategic use of non-specific stressors requires exploiting differences in stress vulnerability between pathogens and hosts. Two basic vulnerabilities of pathogens are: (i) the inherent vulnerability to stress of growth and replication (more immediately crucial for pathogens than for host cells) and (ii) the degree of pathogen localization, permitting the host's use of locally and regionally intense stress. Each of the various types of non-specific stressors is present during severe infections at all levels of localization: (i) ultra-locally within phagolysosomes, (ii) locally at the infected site, (iii) regionally around the infected site and (iv) systemically as part of the acute-phase response. We propose that hosts strategically use a coordinated system of non-specific stressors at local, regional and systemic levels to preferentially harm the pathogens within. With the rising concern over emergence of resistance to specific therapies, we suggest more scrutiny of strategies using less specific therapies in pathogen control. Hosts' active use of multiple non-specific stressors is likely an evolutionarily basic defence whose retention underlies and supplements the well-recognized immune defences that directly target pathogens.

## 1. Introduction

The gold standard of host defences against pathogens is the use of highly targeted effectors that avoid collateral damage to the host. By using molecular differences between pathogens and host cells, the only cost of using specific effectors in defence is their cost of manufacture. Examples include neutralizing antibodies, lysozyme (which targets bacterial cell wall synthesis) and antimicrobial peptides. By contrast, non-specific stressors such as heat, nutrient and oxygen restriction, reactive oxygen and nitrogen species, and acidity can harm pathogens and host cells alike. Nevertheless, many host defences against pathogens involve non-specific stressors that do indeed cause substantial collateral damage. The acute-phase response (APR), which is the systemic response to infection [1,2], has a number of components that are stressful. It has been proposed that hosts use the stressful components of the APR, such as fever and nutrient restriction, to support the more intense stressors at infected sites and to preferentially harm rapidly replicating pathogens [3]. This led us to initially explore the utility and costs of non-specific stress for host defence against pathogens by using an agent-based model of a simple host infected with local generic pathogens [4]. In this review, we further explore and extend the implications of the host's use of non-specific stressors as effectors, and suggest that it is both more prevalent and important than is typically recognized.

We take a broad view of ‘pathogens’ to not only include microorganisms but also infected host cells and tumour cells. We define stress as a disruption of homeostasis, and define stressors as agents causing harm or simply incurring costs, which can be resisted by protective stress responses acting to minimize harm. The costs of stress can include the direct harm by the stressor (e.g. heat damage: loss of function and cost of repair) and the costs to produce the stressor (e.g. energy to produce febrile temperature). However, there are costs of activating protective stress responses (e.g. protein synthesis in the heat shock response) as well as the lost opportunities during protective responses (e.g. disruption of normal protein synthesis and delayed growth and reproduction). Indeed, the most basic protective strategy against the direct harm of stress is to delay growth and replication and to undergo temporary quiescence. In general, organisms facing potential stress are forced to weigh the relative costs of experiencing the stress itself (which may never occur) versus the costs of maintaining reserves and of responding to the expected stress. In addition, hosts must weigh the costs of using stress in defence versus the risks of succumbing to infection due to inadequate defence. An alternative to resisting the pathogens is for the host to have tolerance to manageable numbers of the pathogens, trading the high costs of eliminating every pathogen for the lower continuous costs of pathogen and host harm and risks of pathogen escape from control [5].

Studying non-specific stress for host defence against pathogens may seem like an intellectual backwater in this age of discoveries of the complexity of evolved immune defences and of spectacular biotechnological advances. The use of antibiotics ushered in the age of specific therapy, capitalizing upon molecular differences between bacteria and host cells. Such anti-infective therapy led the way for abandoning primitive non-specific therapies such as inducing malaria to treat syphilis [6] and Coley's toxin, bacterial products that induced immune responses to treat cancers [7]. Unfortunately, their mode of action was never fully elucidated.

The APR likely played a key role in the efficacy of those non-specific therapies. Fever, a well-known component of the APR, has frequently been considered to have provided this non-specific efficacy, but surprisingly there is not even a consensus on how fever acts to protect [3,8,9]. Nor is there consensus on how local heat therapy should be applied to control superficial infections. Which is more important: to stimulate immune responses or to directly harm the pathogens? For instance, should the therapeutic goal be to cause vasodilation using prolonged mild warmth, seeking to stimulate blood flow thereby increasing inflow of nutrients and increasing the functionality of inflammatory cells? Or should the goal be to harm the pathogens, using brief periods of more intense and damaging heat, seeking to avoid the pathogens' protective heat shock responses? In this review, we advocate and provide the rationale for the latter approach of using non-specific stressors as effectors to preferentially harm the pathogens within.

## 2. Strategies for using non-specific stress

There are two fundamental strategies for effectively using non-specific stress for defence: (i) capitalizing on differences in vulnerability and (ii) localizing the stress. Owing to the minor differences between tumour cells and normal cells, it is no coincidence that we find striking similarity between the prominent role of non-specific stress in anti-cancer therapy as developed by clinical researchers [10] and the now obscure, but basic, role of non-specific stress in innate immunity as developed through natural selection.

### (a) Capitalizing on differences in vulnerability

A universal principle is that growth and replication are more vulnerable to stress than is quiescence. Growth and replication require a gathering and splitting of resources, and the process of synthesis typically involves intermediate stages that are more fragile and vulnerable to stress than either the more stable initial or final stages. A vivid example is that a house may well withstand a hurricane, but actually building a house during a hurricane would be folly. Resources (energy and/or materials) that are devoted to growth and replication are not available for withstanding stress. In other words, these resources could have been used for construction of stress-resistant defences, for repair, or for simply surviving until the stress had passed. As expected, cells are most vulnerable to heat and oxidative stress during replication [11–13]; and mitosis, protein synthesis (particularly folding), and ribosome formation are particularly vulnerable to stress [14,15]. Since pathogens typically rely on growth and replication for their pathogenicity, their vulnerability to stress would be expected to be greater than that of the host's cells and of the host itself.

We tested the feasibility of using completely non-specific systemic stress to control simulated localized infections of a simple host in our agent-based model [4]. In the model, aside from the pathogens having the ability to actively harm and gain energy from host cells they were in contact with, the only difference from host cells was that the pathogens replicated faster. We found that applying a uniform stress (energy deprivation) to our simulated host, and to the pathogens within, was an effective but costly and risky means of helping eliminate the pathogens. Essentially, the model confirmed the universal trade-off of using resources either for growth or for resisting stress.

Since the host initiates the defensive stress, it has the advantage of controlling the intensity and timing to inflict damage on the pathogens. Our agent-based modelling showed the value of applying stress periodically. The relief from stress allows the host to partially recover before starting another round of stress. Although this relief from stress also allows the pathogens to grow and replicate again, the pathogens are essentially lured into their most vulnerable state, to be preferentially harmed again once the stress starts anew. An anti-cancer analogue of intermittent stress is systemic chemotherapy given every few weeks to allow the patient to partially recover. We suggest that, if timed well, intermittent stress can also enhance the vulnerability of the target cells.

### (b) Localizing the stress

Localizing the non-specific stress allows for relatively higher intensity at the infected site, thereby causing more harm to pathogens with less harm to distant host cells. As shown in table 1, we describe four categories of spatial localization of stress: (i) *ultra-local*, representing that occurring within phagolysosomes, where the lack of collateral damage essentially meets our definition of specific stress; (ii) *local*, representing the pathogen–inflammatory cell interface at the infected site; (iii) *regional*, the area around the infected site where there is often impaired blood flow due to coagulation (thrombosis), microvascular sludging, extravascular fibrin deposition, oedema and neutrophil extracellular traps (NETs) [22,23] and (iv) *systemic*, the manifestation of the numerous stressful components of the APR.

**Table 1.** Localization and proposed immune utility of non-specific stressors in infection.

stressor localization	reactive oxygen and nitrogen species	heat	reduced glucose, glutamine	reduced Fe, Zn, Mn	reduced oxygen	reduced pH
*ultra-local*	✓	✓	✓	✓	✓	✓
phagolysosomes	respiratory burst	respiratory burst and/or uncoupling proteins^a^	glutaminase degrading glutamine^a^	active uptake from phagolysosomes^a^	used in respiratory burst	active acidification of phagolysosome
*local*	✓	✓^a^	✓	✓	✓	✓
inflammatory by-products	surface respiratory burst, phagosome leakage, mitochondrial generation [16], antioxidant depletion [17]		usage by pathogens and host cells (large amounts by immune cells^a^)	usage by pathogens and binding by lactoferrin and calprotectin^a^	usage by pathogens and host cells	lactic acid as by-product of glycolysis
*regional*	✓	✓	✓	✓	✓	✓
reduced blood flow	reduced influx of antioxidants	reduced removal of local heat				
*systemic*	✓	✓	✓	✓	✓	✓
APR stressors	oxidative stress and decreased antioxidants [18,19]	fever	anorexia, delayed gastric emptying, inhibited gut absorption of sugars and glutamine^a^ [20]	sequestration, reduced absorption of Fe, anorexia	anaemia of inflammation^a^	lactic acidosis [21]

^a^See text for reference.

Table 1 highlights that the same types of non-specific stress, whether primarily destructive or growth inhibiting, tend to occur at each level of localization. Rather than being mere by-products, we propose that each stressor at each level has immune utility. Besides the stress being more intense with greater spatial localization, temporally the non-specific stress begins more locally (i.e. first in the phagolysosomes), such that the more costly systemic stress occurs only if the pathogen cannot be controlled by more localized effectors.

The notion of localizing stress at various levels for defence also has analogues with anti-cancer therapy. Examples at each level are: (i) toxic agents bound to antibodies or liposomes (ultra-local level); (ii) targeted radiation or heat, creams with metabolite inhibitors, surgical excision (local level); (iii) less targeted radiation, regional chemotherapy, angiogenesis inhibitors and wide surgical excision (regional level) and (iv) standard systemic chemotherapy affecting growing and/or replicating cells (systemic level).

## 3. Examples of non-specific stressors for host defence

While table 1 summarizes the primary non-specific stressors used in host defence, it is instructive to describe some of these examples in more depth. We briefly discuss iron restriction, which is well recognized as a stress-based host defence, and heat, which is surprisingly poorly understood in host defence.

### (a) Iron

Iron is a key, and often limiting, nutrient for multiple functions in almost all life forms; and the struggle for iron between microorganisms and hosts is well known [24,25]. Growing and replicating cells have the special problem of satisfying increasing resource needs, and iron restriction can be viewed as a growth-inhibiting stressor. Bacteria and fungi have evolved numerous mechanisms for extracting iron from hosts, most notably the production of siderophores which bind iron for their use. To offset this, neutrophils and macrophages extract iron from phagolysosomes by natural resistance-associated macrophage protein-1 (Nramp1) [26]. Additionally, at the locally infected site, neutrophil-derived lactoferrin binds iron; and regionally, decreased blood flow to the infected site further diminishes iron influx. Finally, at the systemic level, plasma iron concentrations are reduced as part of the APR due to decreased intestinal uptake and sequestration by mononuclear phagocytes. The nutritional deprivation of iron as a non-specific stressor is mirrored by similar mechanisms at the same levels of localization for zinc and manganese, which are also essential micronutrients [26,27], though intriguingly, toxic amounts of zinc may be delivered to phagolysosomes to kill bacteria [28].

### (b) Heat

Analogous to the iron example, fever is a systemic stressor used for host defence, despite the costs [3]. In this viewpoint, fever acts strategically as a stressor in two ways: (i) energy to generate the heat is diverted away from other uses, including away from pathogens and (ii) the heat directly inflicts stress [29,30] as a destructive or damaging stressor by enhancing other sources of heat. However, because febrile temperatures (i.e. core body temperatures) of 39.5–40°C have limited lethality for cultured cells [13,31] or for selected bacterial pathogens in culture [8], and because numerous immune functions are enhanced at slightly elevated temperatures, an opposing view is that the main benefit of fever is not as a stressor to harm pathogens but as an immune stimulant [8]. Unfortunately, heat as a *local* stressor has only rarely been considered [32], and the warmth of inflammation is typically attributed to vasodilation, with the local temperature thereby limited to core body temperature. Nevertheless, local heat is produced by the respiratory burst [33,34], which involves the exothermic reaction of highly reactive oxygen, chlorine and nitrogen intermediates, typically within phagolysosomes. Interestingly, macrophages in arterial plaques have high expression of uncoupling protein 2 (UCP2), which can generate heat at the expense of ATP production [35]. Furthermore, there is direct evidence that inflamed tissues are hotter than core temperatures and that heat is, in fact, locally generated, as demonstrated by the measurement of higher temperatures (up to 2°C) in inflamed atherosclerotic plaques compared with adjacent artery walls [36,37].

Thus, testing of heat sensitivity of pathogens in culture or through artificial whole-body hyperthermia is misleading because: (i) the temperatures to which pathogens are exposed at the infected site are currently unknown and (ii) in culture the pathogens are spared the numerous other inflammatory stressors applied locally, regionally and systemically. Indeed, synergy has been described for heat and iron restriction *in vitro* in killing the pathogenic bacterium, *Pasteurella multocida*, when neither stressor alone was effective [38]. Similarly, synergy of heat and low pH on reducing cell viability has been shown [39]. In addition, although many immune responses are indeed enhanced by slightly elevated temperatures [9,40], rather than being the primary function of fever, this characteristic is instead concordant with the view that immune function evolved to adapt for stressful working conditions, as proposed for improved immune efficacy in slight acidity [41] and slight hypoxia [42]. Therefore, we view the primary evolved purpose of fever, rather than enhancing immune function by expending large amounts of energy, is to act as a systemic stressor, supporting and enhancing the effects of other stressors at all levels of localization in host defence.

## 4. Expectations and implications of using non-specific stress in defence

Once it is recognized that there are differences in vulnerability between pathogens and host cells (and the host as a whole), it is reasonable to propose, as we do here, that hosts evolved to actively enhance stress during infections to preferentially harm the pathogens. From this perspective, we suggest a number of expectations and implications were this to be the case, which are outlined in table 2 and discussed below. We exclude ultra-local stress occurring within phagolysosomes since stress at the ultra-local level has always clearly been a defence and since it does not involve self-harm.

**Table 2.** Expectations and implications of using non-specific stress in defence.

(*a*) local	(i) immune cells should have access to needed resources by (a) bringing along resources and (b) increasing resource uptake locally; (ii) immune cells should make local site stressful by (a) actively depleting resources and (b) generating harmful waste products; (iii) host cell replication should avoid stressful environments;
(*b*) regional	(i) increase stress by reducing blood flow: link inflammation with coagulation;
(*c*) systemic	(i) support regional and local stress with less intensity but wider application;
	(ii) be especially costly since distant host tissue also affected

### (a) Expectations of using local stress for host defence

#### (i) Immune cells should have special access to resources they need

(a) *Immune cells should bring along their own nutrients to the infection site* if feasible. Since neutrophils and macrophages migrate to glucose-limited areas, one might expect that they would bring their supplies with them. Indeed, while these cells contain little glycogen while in the bloodstream, they actively store glucose as glycogen when they migrate to inflammatory sites [43,44].

(b) *Immune cells should increase their local uptake of nutrients at infected sites or when stimulated by inflammatory mediators and pathogen products*, in part to deprive nutrients to pathogens. This increased uptake or utilization by stimulated leucocytes has been shown for glucose [45,46], glutamine [47] and arginine [48]. Borregaard & Herlin [49] found that neutrophils use extracellular glucose in preference to their stored glycogen, further supporting the perspective that they are acting to deprive nearby pathogens of glucose.

#### (ii) Immune cells should actively make the infected site stressful

(a) *Immune cells should actively deplete the nutrients, to the point of being wasteful* by taking in or destroying more nutrients than they can use.

The high and energetically inefficient use of glucose via glycolysis by neutrophils and classically activated (M1) macrophages, even in the presence of substantial oxygen (aerobic glycolysis), has long been noted but not understood [49–51]. Rapidly replicating cells primarily use aerobic glycolysis (the Warburg effect), presumably to divert carbon for biomaterial synthesis [52] or particularly to reduce mitochondrial-derived reactive oxygen species during the vulnerable period of DNA synthesis [11]. However, neither rapid replication nor massive size enlargement is important for immune cells arriving at infected sites. Therefore, the large and apparently inefficient usage of glucose by immune cells at infected sites certainly seems wasteful yet is exactly what is needed to reduce its availability to pathogens.

Glutamine is another key nutrient for host cells and pathogens alike [53,54]. In parallel with the incomplete oxidation of glucose by immune cells, it has been enigmatic as to why neutrophils and macrophages take up far more glutamine than they seem to need, simply storing most of it as lower energy amino acids, notably glutamate, aspartate and alanine [55,56]. Again, we suggest that what appears to be wasteful is indeed deliberately wasteful.

Intriguingly, neutrophil secondary granules have been found to contain glutaminase [57], which converts glutamine to glutamate and ammonia, potentially depleting phagolysosomes of glutamine. Along these lines, arginase I, found in neutrophil primary granules, has been proposed to enhance fungal killing by depleting the phagolysosomes of arginine as a nutrient [58]. Analogous to this is the active local depletion of free iron by binding to locally secreted lactoferrin and of zinc and manganese binding by neutrophil-secreted calprotectin, which are considered host defences based on local nutrient depletion [25,27]. As noted previously, phagocytes can actively remove iron, zinc and manganese directly from their phagolysosomes.

(b) *Immune cells should enhance the formation of harmful waste products at infected sites*. The large usage of glucose through glycolysis has the advantage of causing stress not only by reducing pathogens' access to it, but also by producing correspondingly large amounts of lactic acid, making the infected site even more stressful.

#### (iii) Host cells should avoid replicating and developing at infected sites

Host cells should avoid replicating and developing at infected sites, even though local replication would remove the need for their migration to the site. This avoids the difficulty and risk of replicating under stressful conditions. This contrasts with the difficulty faced by pathogens trying to replicate at the infected site. It is noteworthy that immune effector cells typically replicate and develop at distant sites, such as in the bone marrow, lymph nodes and spleen, places typically not subject to local or regional stress. In accord with this expectation is the recognition that most macrophages at infected sites are bone marrow-derived monocytes [59] and that M1 macrophages, which are aggressively antimicrobial, typically do not replicate locally [60]. By contrast, M2 macrophages, which are involved in the low stress functions of tissue repair and immune suppression, often do replicate locally [60]. A tenet of wound healing is that proper healing first requires resolution of infection/inflammation—infected wounds are stressful, and having proliferating host cells among proliferating pathogens removes the host's advantage of applying stress to help clear the infection.

### (b) Expectations of using regional stress for host defence

#### (i) Infection should be linked with impaired blood flow to increase regional stress

Despite the vasodilation associated with inflammation, the vascular supply to infected areas often becomes impaired. The reduced flow occurs because of the linkage of coagulation with inflammation leading to thrombosis, vascular sludging from adherent leucocytes, fibrin deposition, oedema and intra- and extravascular clogging by NETs. This impaired blood flow around infected sites has been considered to have evolved to reduce the spread of pathogens [22,23,61]. While the impaired blood supply has frequently been noted as contributing to the harsh environment of infected sites, this has been considered an unavoidable by-product of the inflammatory response or pathogen containment. By contrast, we have proposed that, along with restricting pathogen spread, the impaired vascular flow to infected sites also functions to further stress the pathogens therein [4]. We suggest that this impaired vascularity not only impedes the influx of nutrients, oxygen and antioxidants, but also reduces the efflux of lactic acid and locally produced heat, thereby preferentially harming the pathogens. This proposal is supported both by our agent-based model study [4] and common-sense analogies in anti-cancer therapy.

The roles of stress as a defence and pathogen containment are linked in that restricting pathogen spread permits the host to apply more intense stress to the pathogens than would otherwise be feasible. Conversely, uncontained pathogens escape the stress (locally intense and regionally less intense), allowing faster and safer replication.

### (c) Expectations of using systemic stress in host defence

#### (i) Systemic stress of the acute-phase response should be similar to but less intense than local and regional stressors

This similarity would enhance or support more localized stressors while not overburdening distant host cells. By ‘enhance’ we propose, for example, that it is more efficient to keep essential iron from phagocytized pathogens if there is already local depletion of iron at the infected site, which in turn has restricted blood flow (and iron availability) regionally, with the systemic blood also having reduced concentrations of iron. Likewise, the anaemia of inflammation [62], leading to mild hypoxia, may enhance more extreme hypoxia at the infected site.

#### (ii) Systemic stress as a defence should be extremely costly since it affects otherwise untouched host cells distant from the infection site

Because of the importance of this point, we devote the next section to the costs and benefits of systemic stress as a defence.

## 5. Costs and benefits of using systemic stress for host defence

A goal of the field of ecological immunology is to quantitate the costs of resisting infection, since these costs reduce the resources an organism can devote to reproduction, maintenance and surviving environmental challenges. One approach has been to measure the energetic costs associated with activating the various components of the immune response following immunologic challenges (reviewed in [63]). However, others have suggested that these costs related to actually mounting a typical immune response are relatively low in comparison to the major costs associated with mounting a fever, undergoing food restriction or sustaining the collateral self-harm and associated life-history trade-offs [1,64–67]. While local and regional stress can be lethal in critical organ systems (e.g. heart, lungs, brain and kidneys), self-induced systemic stress can be lethal regardless of where the infection is localized. Indeed, sepsis entails the excessive use of systemic defence [68,69]. The costs from the stressful components of the APR come from having to harm all host cells while trying to preferentially harm more localized pathogens. As such, these expensive defences should not be intensively invoked if less costly defences can effectively control the infection [64]. The stressors of the APR should be thought of as the defence of last resort, in which case the costs essentially become irrelevant to a host that would otherwise be killed from pathogen overgrowth.

Our perspective allows us to see the high cost of systemic stress in a positive light and to resolve a key paradox. Why should severe infection be associated with systemic nutrient deprivation, anorexia, delayed gastric emptying, and inhibited absorption of sugars [70] and glutamine [20] just at the time when resources should be needed to fight the infection? Much of the basis of our argument is that the greater sensitivity of pathogens to non-specific stress means that stressing the pathogens as a defence often trumps immediate nourishment of ‘typical’ immune defences. The effective use of the strategy of self-stressing requires adequate resources *before* the infection, as noted by Owen-Ashley & Wingfield [71], who found that birds in poor condition mount a less intense APR (assessed by sickness behaviours such as inactivity and anorexia). The term ‘immune brinksmanship’ was chosen to describe the gamble that the infected host has enough resources at the start of the infection to ‘out-stress’ the pathogens [3]. As suggested by Straub *et al.* [72], the resources should be taken by reprioritization and catabolism of distant tissues for immune defence. Indeed, resources should be directed towards making antibodies, leucocytes, complement components and other acute-phase reactants. However, there are physical limits to the amounts of each of these blood-borne defences that can be used. How can the host put additional remaining resources to use in fighting a severe infection? We propose that the host's production and withstanding of systemic stress to control localized pathogens is an effective means of using the resources of distant host cells. The resources of distant host cells need not be directly transported to immune tissues but can be used by the distant cells themselves to survive the systemic stress to which the entire host subjects itself and the pathogens within. In this view, an infected host with severe clinical cachexia/wasting is likely expending its resources in an appropriately strategic defence. While dying from infection can be considered a tragedy, dying from infection while still having plenty of unused or inaccessible resources would be particularly tragic.

## 6. Conclusion

In the evolutionary arms races between pathogens and hosts, pathogens have evolved a number of ways to avoid and inhibit host defences [19]. However, the multitude of cellular targets of each of several fundamental stressors (e.g. nutrient restriction, heat, reactive molecular species) makes these non-specific stressors a formidable defence, though costly if untargeted. Now that resistance to specific therapies seems to be evolving more rapidly than newer specific therapies can be discovered and developed, the use of non-specific stress should be re-examined. Fruitful areas of inquiry involve exploring interactions among multiple types of stressors, notably destructive stressors and growth-inhibitory stressors, to determine the extent to which they may be synergistic, additive or antagonistic. Finding ideal regimens of timing and intensity are crucial, since stress can either cause significant damage to pathogens when applied rapidly and unexpectedly or can induce protective responses if applied slowly and predictably. In sum, the development of new therapies should consider strategies that exploit the differences in pathogen and host vulnerability to non-specific therapeutics in order to preferentially harm the pathogens.

In the long course of host–pathogen interactions, there has been selection for ever more sophisticated defences against ever more virulent pathogens, leading to the development of the amazingly complex acquired immune system, having memory and high specificity. However, even many of the effectors of the more fundamental innate immune system have specificity (e.g. lysozyme and antimicrobial peptides) against pathogens, which has allowed hosts to use ever more pathogen-damaging defences while avoiding collateral damage. Our intention has been to examine how the use of the most primitive effectors of innate immunity, completely non-specific stressors, play a role in defence. In contrast to the obvious means through which specific stressors can provide defence, in many cases it has not even been apparent how, or even that, the self-harm during infections could provide some degree of host defence. For example, the mode of action of numerous non-specific systemic stressors of the APR has been unclear; and the stressful conditions at and around infection sites are typically regarded as harmful to the host, being unavoidable by-products of the host–pathogen struggle, without consideration that the pathogens may be disproportionately harmed. In retrospect, the efficacy of non-specific stressors, actively applied by the host locally, regionally and systemically, makes sense. After all, the same principles are used in therapy against tumour cells, pathogenic cells differing from normal host cells primarily in growth rate and their degree of localization. Indeed, the strategy of using non-specific stress to capitalize on differences in vulnerability between contestants has universal application. We propose that to the extent that hosts can harm themselves to preferentially harm the pathogens within, we should expect hosts to actively use this strategy as a defence.
